# Piezoresistive Effect of Conductive and Non-Conductive Fillers in Bi-Layer Hybrid CNT Composites under Extreme Strain

**DOI:** 10.3390/ma16186335

**Published:** 2023-09-21

**Authors:** Won-Jin Kim, Kun-Woo Nam, Byung-Ho Kang, Sung-Hoon Park

**Affiliations:** Department of Mechanical Engineering, Soongsil University, 369 Sangdo-ro, Dongjak-gu, Seoul 06978, Republic of Korea; dnjswls0214@gmail.com (W.-J.K.); kwn1522@naver.com (K.-W.N.); royce2080@naver.com (B.-H.K.)

**Keywords:** carbon nanotube, polymer composite, piezoresistive effect, secondary filler

## Abstract

Polymers mixed with conductive fillers hold significant potential for use in stretchable and wearable sensor devices. Enhancing the piezoresistive effect and mechanical stability is critical for these devices. To explore the changes in the electrical resistance under high strains, typically unachievable in single-layer composites, bi-layer structures were fabricated from carbon nanotubes (CNTs) and EcoFlex composites to see unobservable strain regions. Spherical types of non-conductive fillers composed of polystyrene and conductive filler, coated with Ni and Au on non-conductive fillers, were used as secondary fillers to improve the piezoresistive sensitivity of composites, and their respective impact on the conductive network was compared. The electrical and mechanical properties were examined in the static state to understand the impact of these secondary fillers. The changes in the electrical resistance under 100% and 300% tensile strain, and their dependence on the inherent electrical properties of the secondary fillers, were also investigated. Single-layer CNT composites proved incapable of withstanding 300% strain, whereas the bi-layer structures proved resilient. By implementing cyclic stretching tests, contrary to non-conductive fillers, reduced piezoresistive influence of the conductive secondary filler under extreme strain conditions could be observed.

## 1. Introduction

Stretchable devices and wearable strain sensors are garnering increasing interest across diverse sectors, ranging from healthcare to robotics [1,2,3,4,5]. Traditionally, such devices have relied heavily on metal-based materials or semiconductors integrated with micro/nanostructures [6,7,8]. Despite the superior electrical and mechanical properties of these materials, they present restrictions in durability, elasticity, and reusability. Consequently, contemporary research trends are pivoting towards nanomaterials to overcome these hurdles, with carbon nanotubes (CNTs) emerging as strong contenders due to their remarkable electrical conductivity and mechanical properties [9,10,11]. CNT/polymer composites, exhibiting both elasticity and electrical conductivity, are positioned as potential substitutes for conventional metal-based materials [9,12].

The piezoresistive effect is characterized by variations in the electrical resistance of a material in response to external stimuli such as compression or tension. Thus, piezoresistivity is a vital mechanism in stretchable devices and wearable strain sensors, so research involving a wide range of materials exhibiting piezoresistive effects is continuously advancing [13,14,15,16]. The piezoresistive effect of the polymer/CNT composite mentioned above is greatly affected by the filler’s size, type, and quantity [17,18]. Numerous studies have examined the piezoresistive effects in CNT composites, with a focus on observing the changes in the electrical resistance when a secondary filler is integrated into the composite [5,19,20]. Although these studies propose techniques for enhancing the piezoresistive effect, most fail to capture resistance changes under considerable deformation due to the constraints imposed by the single-layer structure.

The process of fabricating single-layer composites is relatively straightforward; nevertheless, such materials are prone to significant plastic deformation under external forces such as stretching and compression [21,22]. Furthermore, higher filler contents adversely affect the elongation rate (inverse relationship) [17,23,24]. To address these challenges, herein, we introduce bi-layer CNT composites by attaching an elastomer layer under single-layer structure composites [25,26,27]. In fabricating these bi-layer CNT composites, a silicone rubber (EcoFlex^®^) is used as the lower substrate, onto which an EcoFlex/CNT composite paste is applied in a 6:1 thickness ratio. The structure is formed via heat pressing, and the successful integration of the two layers is verified through optical microscopy.

The mechanical properties and electrical conductivity of the respective composites are comparatively analyzed. Furthermore, to scrutinize the piezoresistive effect, the changes in the electrical resistance are monitored under 100% and 300% strain. The observations reveal that the single-layer composites could not endure 300% strain and ruptured at the maximum strain point, in contrast with the bi-layer structure. In essence, this study presents a method of fabricating bi-layer CNT composites and proposes a technique for fine-tuning the piezoresistivity of CNT composite sensors or devices.

## 2. Materials and Methods

### 2.1. Materials

Multi-wall carbon nanotubes (MWCNTs) were used as the primary conductive fillers, while spherical particles served as the secondary fillers. The MWCNTs, with an average outer diameter of 6 nm and a length range of 50–150 μm, were purchased from JEIO (Incheon, Republic of Korea). Two types of spherical particles (H5.0 bead for non-conductive and PGM H5.0R for conductive) with an average diameter of 5 μm were purchased from Duksan Hi-Metal (Ulsan, Republic of Korea). The composition of the non-conductive fillers, H 5.0 bead, is polystyrene; and the conductive secondary fillers, PGM H5.0R, are coated with Ni and Au on the surface of the polystyrene-based non-conductive fillers. Silicone rubber (EcoFlex 00-30^®^), a polymer, was acquired from Smooth-On Inc. (Macungie, PA, USA) and used as the matrix material.

### 2.2. Fabrication of Single and Bi-Layer Composites

During the fabrication of the bi-layer composites, the fillers were dispersed within the matrix using a paste mixer (Daehwa, Seoul, Republic of Korea) and a 3-roll milling machine (Intec, Gyeonggi-do, Republic of Korea). For the upper layer paste, EcoFlex A (premixing elastomer) and EcoFlex B (curing agent) were combined in a 1:1 mass ratio, followed by the addition of the MWCNTs and secondary fillers at respective concentrations of 1 wt% and 25 vol%. Secondary fillers were used in different pastes to achieve conductivity and non-conductivity, respectively. The paste was mixed at 500 rpm for 30 s, followed by additional mixing at 1500 rpm for 90 s. The paste was then treated with a 3-roll milling machine for 90 s to achieve homogenous dispersion of the fillers in the paste.

**Figure 1:**
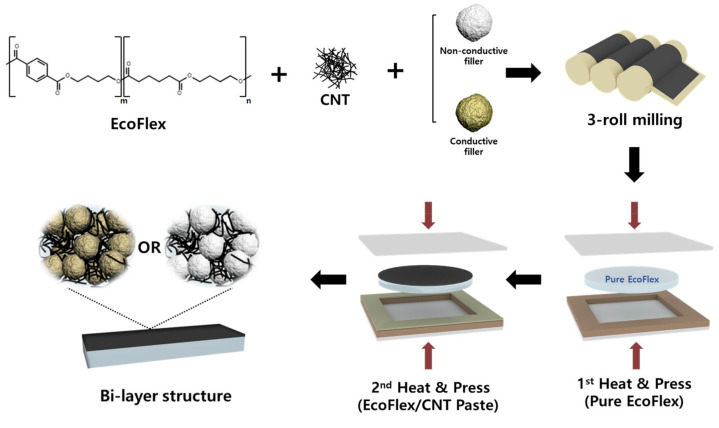
Fabrication of bi-layer CNT composites with spherical secondary fillers: conductive and non-conductive.

EcoFlex A and B were mixed in a 1:1 mass ratio using a paste mixer to make the lower substrate. The mixture was poured into a 150 mm × 150 mm × 12 mm mold, and air bubbles were removed by keeping the mold in a vacuum oven at room temperature for 15 min (SH Scientific Co., Sejong, Republic of Korea). The treated EcoFlex was partially cured by passing through a hot press machine (Limotem, Gyeonggi-do, Republic of Korea) at 55 °C and 15 bar for 20 min. Subsequently, the prepared paste was placed in the center of the substrate, with a 150 mm × 150 mm × 0.2 mm mold overlapping it, and then cured at 150 °C and 15 bar for 1 h to obtain the bi-layer composites. As a comparative group, single-layer and bi-layer composites without secondary fillers were produced under the same conditions as mentioned above. The single-layer composite was cured without substrates using a 150 mm × 150 mm × 0.2 mm mold. This process is represented in Figure 1.

### 2.3. Characterization and Test Conditions

The layer-by-layer thickness and morphology of the bi-layer composites were observed and analyzed using a LEXT OLS5000 confocal microscope (Olympus, Shinjuku, Japan) and a Gemini SEM 300 (ZEISS Inc, Land Baden-Württemberg, Germany) instrument. The thickness was measured from the cross-section of a 5 mm × 1 mm sample, and the dispersity of the fillers in the composites was analyzed by observing the fracture surface of the specimens. The fracture was obtained by freezing a 50 mm × 5 mm sample with liquid nitrogen, followed by fracturing. 

To measure the maximum tensile range of the composite, the specimen was manufactured according to the ASTM D638-5 standard [28] and analyzed using a universal testing machine (UTM; DRTECH, Gyeonggi-do, Republic of Korea). Six samples were tested using a 20 kgf load cell at a testing speed of 20 mm/min, and an average value was derived.

To measure the electrical conductivity of the composites and the resistance change during the tensile tests, the composite was cut to a specific size to form an electrode. The composite was cut to dimensions of 50 mm × 5 mm and treated with a UV-ozone cleaner (JSE Co., Seoul, Republic of Korea) for 5 min to maximize the exposure of CNTs on the surface to facilitate the subsequent step, i.e., electrode fabrication. Thereafter, silver paste (Protavic, Daejeon, Republic of Korea) was applied to both ends of the specimen and cured at 150 °C for 1 h. The electrical conductivity of the composites in the static state was measured using a Fluke 114 multimeter (Fluke, Washington, USA). The resistance changes during the tensile tests were analyzed using a Keithley DMM 7510 multimeter (Keithley, Cleveland, OH, USA) and a three-dimensional tensile machine (Namil, Incheon, Republic of Korea).

## 3. Results and Discussion

### 3.1. Morphology Analysis

**Figure 2:**
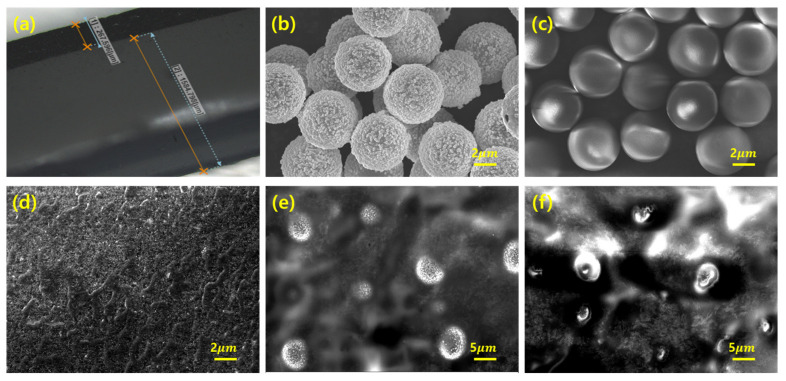
(**a**) Confocal microscopy image showing thickness of each layer. The upper layer is the CNT composite, and the lower layer is the EcoFlex substrate. SEM images of (**b**) conductive fillers, (**c**) non-conductive fillers, (**d**) bi-layer CNT composite without any secondary filler, (**e**) with conductive fillers, and (**f**) with non-conductive fillers.

Figure 2a presents a confocal microscopy image of the cross-section of the bi-layer composite. Figure 2b,c show the morphology of the secondary fillers, and Figure 2d–f illustrate the fracture surface of the bi-layer composites. Figure 2a shows the thickness of each upper and lower layer in the bi-layer composites. The substrate was fabricated by utilizing a 1.2 mm mold; the upper layer was 0.2 mm thick. The thickness ratio of the upper and lower layers did not deviate significantly from the mold thickness ratio of 6:1, indicating that each layer was firmly bonded.

Figure 2b shows the conductive spherical polymers coated with nickel and gold. Figure 2c shows the non-conductive spherical polymers without any coated metal. Both particles had an average diameter of 5 µm. The conductive particles had a chestnut-like appearance, whereas the non-conductive particles were almost completely sphere-like. Figure 2d–f show the dispersity of the fillers in the upper layer. It is evident that both the MWCNTs and the secondary filler were uniformly dispersed throughout the composites by employing the paste mixer and 3-roll milling machine [29].

### 3.2. Mechanical and Electrical Properties

**Figure 3:**
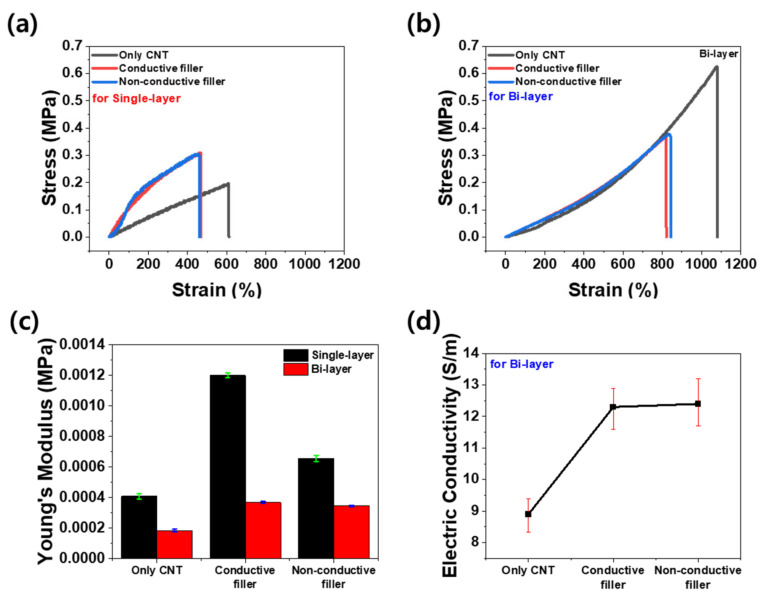
Strain–stress curves of (**a**) single-layer CNT composites, (**b**) bi-layer CNT composites, (**c**) Young’s modulus of single-layer composites (black bar) and bi-layer composites (red bar), and (**d**) electrical conductivity of bi-layer CNT composites according to the type of secondary filler.

Figure 3a,b show the strain–stress curves of the single-layer and bi-layer composites. Both graphs show that the composites incorporating the secondary fillers underwent less elongation compared to the composites without secondary fillers. To enhance the strain of the composite with secondary fillers, we developed a bi-layer structure by bonding a polymer substrate underneath [22]. The resulting strain–stress curves of the bi-layer composites are illustrated in Figure 3b. The tensile strain in the bi-layer composite consisting of the substrate and the composite at a thickness ratio of 6:1 was approximately twice the maximum of that in the single-layer composite. As a result, the effect of the secondary fillers on the high-elongation composite could be analyzed, which cannot be accomplished in the single-layer composite. Figure 3c presents a bar graph showing the Young’s modulus of all the composites in Figure 3a,b. Notably, the Young’s modulus of the single-layer and bi-layer composites varied by a factor of 2 to 3 depending on the filler composition. The introduction of secondary fillers contributed to an overall increase in the Young’s modulus for the single- and bi-layer composites by increasing the brittleness of the composites [30]. In the case of the single-layer composites, the Young’s modulus was approximately twice as large when secondary conductive fillers were incorporated compared to secondary non-conductive fillers. This difference is due to the hardness of the conductive filler resulting from the nickel and gold plating, while the non-conductive filler is relatively softer as it lacks plating [31,32]. However, for the bi-layer composite, the type of secondary filler did not lead to a significant difference in the Young’s modulus because the substrate of the bi-layer significantly reduced the extent to which the stress in the composite increased due to the presence of the secondary filler. Figure 3d illustrates the electrical conductivity of the bi-layer composites in the static state. The electrical conductivity was calculated as follows:

(1) $$\sigma_{conductivity}=\frac{l}{wtR}$$

where $\sigma_{conductivity}$ is the electrical conductivity, *l* is the length of the specimen, *R* is the electrical resistance of the specimen, *w* is the width of the specimen, and *t* is the height of the specimen. As electrons only flow on the upper layer, the upper thickness of the bi-layer composite was used as the height (*t*) of the specimen. The average electrical conductivity was determined as the average for six samples (50 mm × 5 mm) within a single composite. The electrical conductivity of the composite exclusively comprising MWCNTs as fillers was 8.9 S/m. However, the electrical conductivity of the composite containing conductive particles as a secondary filler was 12.3 S/m and that of the composite containing non-conductive particles was 12.4 S/m. These results reveal two significant phenomena. First, the composites containing micro-scale secondary fillers have a higher electrical conductivity compared to those composed of only CNTs. This enhancement can be attributed to the excluded volume effects [33,34,35]. The inclusion of micro-scale particles into the composite reduced the available space for CNTs, resulting in increased contact between the CNTs. Consequently, the electrical connections were improved, leading to enhanced electrical conductivity. Second, even if the secondary filler was conductive, it had little effect on the electrical conductivity of the composite compared to the non-conductive secondary filler. Negligible differences were observed between the composites employing conductive particles as secondary fillers and those incorporating non-conductive particles. As illustrated in Figure 2e,f, including a 25% volume fraction of secondary fillers did not facilitate particle connection. Hence, the electrical connections were dominated by the interconnections between the CNTs.

### 3.3. Piezoresistive Properties

**Figure 4:**
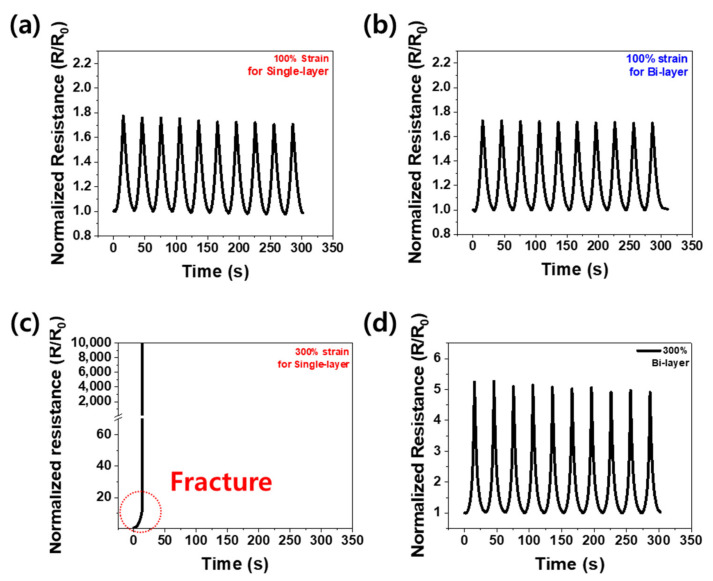
Normalized resistance change (*R*/*R*_0_) during 10 cycles: (**a**) at 100% strain for single-layer composites, (**b**) at 100% strain for bi-layer composites, (**c**) at 300% strain for single-layer composites, and (**d**) at 300% strain for bi-layer composites.

The piezoresistivity of the composites was analyzed through tensile tests to understand the effect of structural differences between the single-layer and bi-layer. Figure 4a,b show the relative change in resistance (*R*/*R*_0_) of the single-layer and bi-layer composites using only CNTs as primary fillers under 100% cyclic tensile deformation (*R* is the real-time electrical resistance of the composite; *R*_0_ is the initial electrical resistance of the composite). Each sample was subjected to 10 pre-strains to obtain a steady value, after which *R*/*R*_0_ was determined according to the tensile deformation [36,37]. Under 100% strain, there was only a little difference in the change in the electrical resistance of the single versus bi-layer composites. Thus, it is assumed that if the fraction of the matrix and the fillers (wt%) is equivalent, the volume of the electrical path would be the same in both the single- and bi-layer composites. Figure 4c,d show *R*/*R*_0_ from the cyclic tensile strain tests under 300% strain for the single-layer and bi-layer composites, respectively. For the single-layer composite, the graph shows an infinite rise at the start of 15 s, which is the maximum stretching point, indicating that the composite was fractured during the test, resulting in an infinity resistance value. On the other hand, for the bi-layer composite, the graph shows a stable *R*/*R*_0_ value even under the cyclic tensile strain of 300%. This means that the single-layer cannot withstand 300% tensile deformation, unlike the bi-layer, which is in good agreement with the data in Figure 3c, which shows that the maximum tensile range of the bi-layer is greater than that of the single-layer.

**Figure 5:**
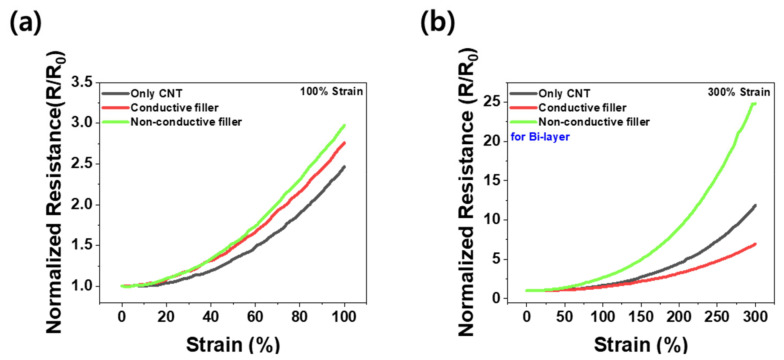
Variation of normalized resistance (*R*/*R*_0_) with strain for bi-layer composites at (**a**) 100% strain and (**b**) 300% strain.

Figure 5 illustrates the variation in the normalized electrical resistance with the different types of secondary fillers. Each test was performed without any pre-strain cycle. As demonstrated in Figure 5a, under 100% strain, the maximal normalized resistance (*R*/*R*_0_) was highest for the composites with non-conductive fillers, followed by those with conductive fillers, and lowest for the composites without any secondary fillers. These differences are attributed to the type of secondary filler employed. The inclusion of micro-scale secondary fillers induces an increase in the electrical resistance by disrupting the electrical path [5,20]. Furthermore, the composites with conductive secondary fillers exhibited smaller *R*/*R*_0_ values than those with non-conductive secondary fillers, suggesting that the conductive secondary fillers contribute to the electrical path. However, this effect is not prominent enough to lower the *R*/*R*_0_ value relative to that of the composites devoid of secondary fillers. This result suggests that 100% strain is insufficient to adequately observe the effects of the conductive fillers. On the other hand, as depicted in Figure 5b, under 300% strain, although the *R*/*R*_0_ values remained the highest for the composites with non-conductive fillers, the *R*/*R*_0_ values of the composites with conductive fillers were lower than those of the composites with only CNTs. This observation emphasizes that under significant deformation (300% strain in this study), the conductive secondary fillers exert a considerable influence on the electrical network, unlike the case at 100% strain.

**Figure 6:**
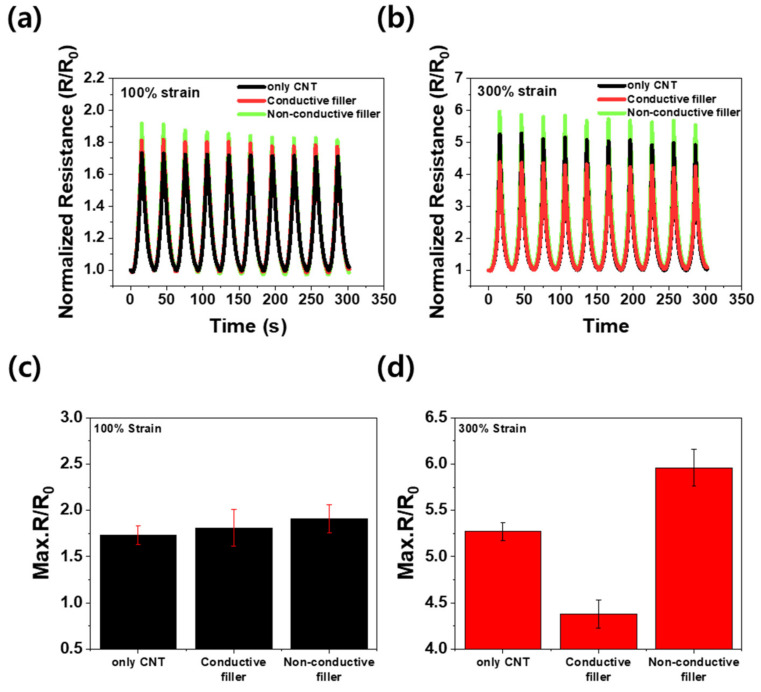
Normalized resistance change (*R*/*R*_0_) over 10 cycles for bi-layer CNT composites: (**a**) at 100% strain and (**b**) 300% strain. Maximum resistance change: (**c**) at 100% strain and (**d**) 300% strain.

Figure 6a,b show the *R*/*R*_0_ values of the bi-layer composites based on the type of secondary filler under 100% and 300% cyclic tensile strain, respectively. The *R*/*R*_0_ of the composites surged to a maximum value at the highest tensile strain, then decreased to a minimum value when the composite was returned to its original state. Each composite was subjected to ten pre-strain cycles before the experiments to ensure stable signals. As shown in both graphs, the composites demonstrated stable resistance changes under tensile strains of 100% and 300% subsequent to applying the pre-strain. As displayed in Figure 6c, under 100% tensile strain, the maximum *R*/*R*_0_ of the composite with the non-conductive secondary fillers was 0.18 higher than that of the composite containing only CNTs, and the maximum *R*/*R*_0_ of the composite consisting of conductive secondary fillers was 0.08 higher than that of the composite made only of CNTs. However, as shown in Figure 6d, the maximum *R*/*R*_0_ of the composite with non-conductive secondary fillers at 300% tensile strain was as high as 0.69 compared to that of the composite with CNTs only, whereas the maximum *R*/*R*_0_ of the composite with conductive secondary fillers fell by 0.89 compared to that of the composite with solely CNTs.

Comparing this outcome with the results of the tensile test in Figure 5, which did not involve pre-strain, the maximum *R*/*R*_0_ difference between the composites declined due to alignment of the CNTs within the composite when the pre-strain was applied. Consequently, the trend in the resistance change of the composites containing conductive and non-conductive secondary fillers was similar under 100% and 300% tensile strain, unlike the case for the composite consisting only of CNTs.

**Figure 7:**
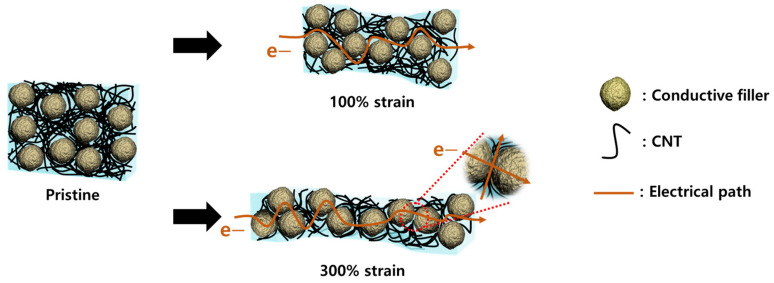
Schematic of CNTs and conductive secondary fillers within composites at 100% and 300% strain. Electrons can move only through CNTs at 100% strain but can also move through secondary fillers at 300% strain.

Figure 7 presents a schematic of the position of the CNTs and secondary fillers within the composites under static conditions, as well as under 100% and 300% tensile deformation. In the static state, there is no interaction between the secondary fillers; thus, the electrical connection is established solely with the CNTs. Additionally, the presence of secondary fillers promotes CNT aggregation via the excluded volume effect, thereby increasing electrical conductivity, as depicted in Figure 3c. Under 100% strain, the electrical resistance increases as the separation between the CNTs expands, whereas the slight change in the distance between the secondary fillers has no effect on the electrical connection.

Nevertheless, under the substantial tensile strain of 300%, the positioning of the fillers changes significantly. The previously established electrical connection is lost as the distance between the CNTs expands in the tensile direction. Simultaneously, the secondary fillers drift apart along the tensile axis but approach each other perpendicularly [38,39]. As a result, the contact between the CNTs and secondary fillers becomes stronger, leading to the observation of two additional phenomena. When the secondary filler particles are non-conductive and spherical, they act as ‘barriers’ under considerable elongation, further hindering the connection of the CNTs, eventually inducing a massive change in the electrical resistance. In contrast, if the secondary fillers are conductive, they mitigate the change in the electrical resistance by acting as a ‘glue’ that reconnects the separated CNTs under extreme tension.

## 4. Conclusions

To investigate the changes in the electrical resistance under significant tensile strain that cannot be achieved in a single-layer composite, a bi-layer structure was introduced into the CNT/EcoFlex composites with secondary fillers. The experiments revealed that the bi-layer exhibited a lower Young’s modulus and a higher elongation compared to the single-layer. Furthermore, in the static state, the inclusion of secondary fillers resulted in a similar increase in conductivity, irrespective of the type of filler. This can be attributed to the excluded volume effect of the secondary fillers, which reduces the space that the CNTs can occupy, thereby facilitating aggregation between the CNTs. Moreover, the study examined the variation in the electrical resistance based on the electrical characteristics of the secondary filler under 100% and 300% tensile strain. Under 100% strain, the influence of the secondary fillers on the resistance changes in the composites was negligible. However, under 300% strain, the resistance changes in the composites exhibited a substantial dependence on the electrical properties of the secondary fillers. In addition to the separation of the CNTs due to tension, the distance between the spherical non-conductive particles was reduced under large tensile strain, seriously interfering with the flow of electrons. Conversely, the inclusion of conductive fillers offset the reduced electrical connection caused by the increased distance between the CNTs, as the conductive particles that moved closer together mitigated the change in the electrical resistance. In conclusion, the electrical resistance of CNT composites having a bi-layer structure with conductive fillers changes to a lesser degree during extreme stretching, compared to that in the single-layer congener. This research defines one criterion for the selection of secondary fillers aimed at enhancing the performance of CNT composites.

## Data Availability

Not applicable.
